# Metal Nanoparticles Released from Dental Implant Surfaces: Potential Contribution to Chronic Inflammation and Peri-Implant Bone Loss

**DOI:** 10.3390/ma12122036

**Published:** 2019-06-25

**Authors:** Eriberto Bressan, Letizia Ferroni, Chiara Gardin, Gloria Bellin, Luca Sbricoli, Stefano Sivolella, Giulia Brunello, Devorah Schwartz-Arad, Eitan Mijiritsky, Miguel Penarrocha, David Penarrocha, Cristian Taccioli, Marco Tatullo, Adriano Piattelli, Barbara Zavan

**Affiliations:** 1 Department of Neurosciences, University of Padova, 35133 Padova, Italy; eriberto.bressan@unipd.it (E.B.); luca.sbricoli@unipd.it (L.S.); stefano.sivolella@unipd.it (S.S.); giulia-bru@libero.it (G.B.); 2 Maria Cecilia Hospital, GVM Care & Research, 48033 Cotignola (RA), Italy; letizia.ferroni@gmail.com (L.F.); chiaragardin@gmail.com (C.G.); gloria.bellin@gmail.com (G.B.); 3 Department of Management and Engineering, University of Padova, 36144 Vicenza, Italy; 4 Schwartz-Arad Day-Care Surgical Center, Maxillo-Facial Surgery, Advanced Implantology, Periodontology & Endodontology, 5219100 Ramat Hasharon, Israel; dubi@schwartz-arad.co.il; 5 Deptartment of Otolaryngology, Head and Neck and Maxillofacial Surgery, Tel-Aviv Sourasky Medical Center, Sackler Faculty of Medicine, Tel-Aviv University, 5219100 Ramat Aviv, Israel; mijiritsky@bezeqint.net; 6 Oral Surgery and Implantology, Valencia University Medical and Dental School, 46004 Valencia, Spain; Miguel.Penarrocha@gmail.com (M.P.); david.penarrocha@gmail.com (D.P.); 7 Department of Animal Medicine, Health and Production, University of Padova, 35133 Padova, Italy; cristian.taccioli@unipd.it; 8 Stem Cells Unit, Marelli Health, Tecnologica Research Institute, Stem cell Unit, 88900 Crotone, Italy; marco.tatullo@tecnologicasrl.com; 9 Department of Medical, Oral and Biotechnological Sciences, University of Chieti-Pescara, 66145 Chieti, Italy; adriano.piattelli@unich.it; 10 Department of Biomedical Sciences, University of Padova, 35100 Padova, Italy

**Keywords:** titanium nanoparticles, reactive oxygen species, osteolysis

## Abstract

Peri-implantitis is an inflammatory disease affecting tissues surrounding dental implants. Although it represents a common complication of dental implant treatments, the underlying mechanisms have not yet been fully described. The aim of this study is to identify the role of titanium nanoparticles released form the implants on the chronic inflammation and bone lysis in the surrounding tissue. We analyzed the in vitro effect of titanium (Ti) particle exposure on mesenchymal stem cells (MSCs) and fibroblasts (FU), evaluating cell proliferation by MTT test and the generation of reactive oxygen species (ROS). Subsequently, in vivo analysis of peri-implant Ti particle distribution, histological, and molecular analyses were performed. Ti particles led to a time-dependent decrease in cell viability and increase in ROS production in both MSCs and FU. Tissue analyses revealed presence of oxidative stress, high extracellular and intracellular Ti levels and imbalanced bone turnover. High expression of ZFP467 and the presence of adipose-like tissue suggested dysregulation of the MSC population; alterations in vessel morphology were identified. The results suggest that Ti particles may induce the production of high ROS levels, recruiting abnormal quantity of neutrophils able to produce high level of metalloproteinase. This induces the degradation of collagen fibers. These events may influence MSC commitment, with an imbalance of bone regeneration.

## 1. Introduction

Several publications have indicated that dental implants are a predictable option for the replacement of missing teeth, due to their high survival and success rates [1]. At the same time though, it has been demonstrated that there is an increased prevalence of biological complications (i.e., peri-implant mucositis and peri-implantitis) [2]. Peri-implant mucositis is defined by the American Academy of Periodontology as an inflammatory disease involving the soft tissues in absence of peri-implant bone loss, while in peri-implantitis the inflammation involves both hard and soft tissues and a progressive loss of the supporting bone is present [3,4,5,6]. The use of different thresholds referring to probing depth and radiographic bone loss for defining peri-implant diseases gives rise to a considerable variability in the reported prevalence of peri-implant disease [7]. In implant dentistry is it well accepted that a degradation process at the implant-abutment connection could be favored by several factors, such as the presence of a biofilm that, acting as lubricant, decreases the friction on the titanium (Ti) surfaces and causes micro-movements, which, in turn, can lead to wear. Moreover, biofilms can alter the pH, inducing wear particle formation as well. In vitro tests confirmed that micro-movements occurring at the implant-abutment connection can increase the wear of the inner surfaces of the connection and, subsequently, the release of metal ions and micro- and nanoparticles into the surrounding tissues [8,9,10,11]. In this view, the main question is if these degradation products released from dental implants could affect peri-implant tissue, inducing pathologic bone resorption [8]. It is well accepted that metal nanoparticles are able to induce inflammatory effects through their immunomodulatory capacity, mainly exerted at a macrophage level [8,12], by means of an increase in DNA damage, protein carbonylation, lipid peroxidation, oxidative stress and by a decrease in the activity of superoxide dismutase, total glutathione levels, and total antioxidant capacity catalase. Moreover, they induce an abnormal activation state of macrophages characterized by an excessive inflammation and a suppression of innate immune function [13,14].

In a recent work of Oliveira and co-workers published in 2018 [8], a review of the literature on the effects of implant corrosion products on peri-implant tissues was performed. The results showed the activation of the osteoclasts activity in presence of metal particles and ions, the presence of macrophages that phagocytosed Ti microparticles and mutations in human cells cultured in medium containing Ti-based nanoparticles. The authors concluded that debris released from the degradation of dental implants had cytotoxic and genotoxic potential for peri-implant tissues. In this view also Fretwurst et al. recently affirmed [15] that we cannot escape from asking ourselves if metal and Ti particles that can be detected in peri-implant supporting tissues could have a local and/or systemic impact on the peri-implant soft and hard tissues. In light of these considerations, it was here analyzed the presence of metal nanoparticles in peri-implant tissue of sites affected by peri-implantitis, their concentration and distributions. Moreover, in vitro study was performed aiming to assess the effect of Ti nanoparticles on mesenchymal stem cell (MSC) physiology, reactive oxygen species (ROS) production and commitment onto osteogenic and adipogenic phenotypes. In tissue affected by peri-implatitis, the presence of metal nanoparticles was investigated and the morphology of the extracellular matrix and of the vessels was analyzed. Moreover, we analyzed gene expression of markers related to the bone lysis observed on tissues affected by peri-implantitis, such as remodeling enzyme, osteogenic osteoblastic markers and gene involved in bone healing. Particular attention was focused on the speculation whether a chronic inflammatory environment could affect the osteogenic commitment of MSCs inducing and alternating bone homeostasis, finally leading to bone lysis.

## 2. Materials and Methods

### 2.1. Patient’s Selection

In this cross-sectional study, a total of 200 non-smoker individuals, aged 30–50 years old, were recruited: 160 patients affected by peri-implantitis (test group) and 40 individuals not affected by peri-implant disease (control group). The subjects were recruited among patients who had attended the Dental Clinic of University of Padova (Italy), the Schwartz-Arad Day-Care Surgical Center Ramat (Israel), the School of Dental Medicine of Tel-Aviv University Ramat Aviv (Israel), and the University Medical and Dental School of Valencia (Spain). The Ethical Committee of Padua Hospital approved the research protocol. All individuals had provided informed consent to be involved in the study, and the study was conducted in compliance with the Declaration of Helsinki ethical guidelines [16].

The study used a convenience sample because it was designed as a hypothesis generator. The sample size of each test was driven by practical reasons (including costs, time, staff workload, and resources).

The exclusion criteria were as follows: (a) ongoing orthodontic therapy; (b) systemic disorders, such as diabetes mellitus, hepatitis, HIV infection, and chemotherapy; (c) pregnancy and lactation; (d) drug administration during the past three months; (e) history of bisphosphonates, monoclonal antibodies, high dosage corticosteroids therapy, or radiotherapy of the cervicofacial district.

The inclusion criteria for the group affected by peri-implantitis were as follows: (a) presence of one or more implants with a minimum loading period of 12 months; (b) peri-implant probing pocket depth > 5 mm; (c) peri-implant presence of bleeding on probing (with/without suppuration); (d) radiographic signs of crestal bone loss in at least one area around an implant; and (e) exposure of at least two implant threads. The control group consisted of subjects undergoing dental implant positioning who (a) had no history or clinical signs of periodontitis or peri-implantitis; (b) had a probing pocket depth equal or less than 4 mm; and (c) had no radiographic signs of peri-implant bone resorption.

In the test group, circumferential peri-implant soft tissue samples were collected during resective surgical treatment of peri-implantitis or in case of extraction of failed implants due to peri-implant disease. In the control group, semi-submerged healing was chosen and the retrieved specimen of mucosa, allowing the positioning of a healing abutment of larger diameter at the second stage after 8–12 weeks from implant placement, was collected instead of being discharged as waste material as usual.

After classifying the two groups according to the mentioned criteria, blood samples (5 cc) were obtained from the arm vein in 30 patients with peri-implantitis and in 20 patients of the control group and collected into tubes containing EDTA (etilen-diammino-tetraacetic). In order to conduct the laboratory tests in a blinded manner, all tubes were assigned a unique subject-specific code.

**Figure 1:**
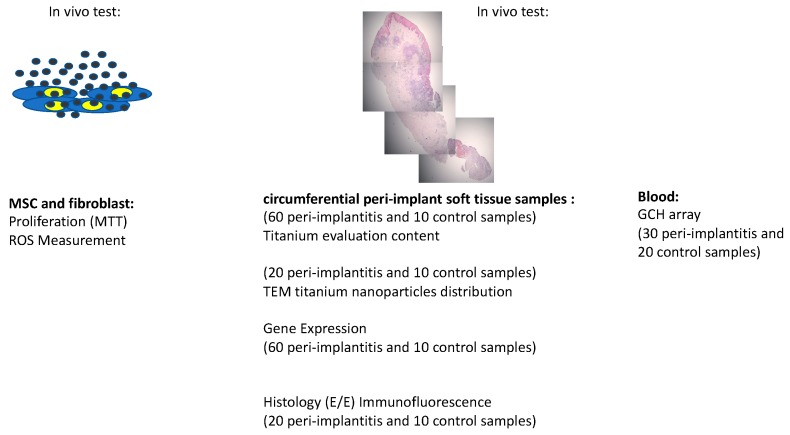
Design of the study.

Design of the study is resumed here follow (Figure 1):

### 2.2. Particulate Preparation and Characterization

Commercially pure titanium oxide microparticles (size distribution: 0.2–0.8 μm; median: 0.488 μm; mode: 0.426 μm; Sigma-Aldrich, St. Louis, MO, USA) were treated to remove >99.94% of adherent endotoxins [17]. After passivation (25% nitric acid wash, 70 °C, 1 h), the particles were washed 3 times with sterile phosphate-buffered saline (PBS; EuroClone, Milano, Italy) and sterilized in 70% ethanol for 30 min at room temperature. Next, the particles were incubated for five alternating cycles in 0.1 M NaOH/95% ethanol (30 °C, 20 h) and 25% nitric acid (room temperature, 20 h) and were washed with sterile PBS between each incubation. After three additional washes with sterile PBS, the particles were re-suspended in cell culture medium consisting of high-glucose Dulbecco’s Modified Eagle’s Medium (DMEM; EuroClone), 10% Fetal Bovine Serum (FBS; EuroClone), and 1% penicillin/streptomycin/amphotericin B (Sigma-Aldrich) (complete DMEM, cDMEM). Particles were counted with a coulter, and the suspension was stored at 4 °C. Prior to use, the particle suspensions were warmed to 37 °C.

### 2.3. Cell Cultures

Human adult dermal fibroblasts (FU) were purchased (Sigma-Aldrich). MSCs were isolated from human dental pulp extracted from healthy molar teeth, removed for orthodontic reasons or for recurrent mucosal pericoronitis. Each subject gave informed written consent for the use of the so obtained dental pulps. The Ethical Committee of Padua Hospital approved the research protocol. Before extraction, each subject was checked for systemic and oral infections or diseases. Only disease-free subjects were selected for pulp collection.

Stem cell isolation was performed according to our previously published protocol [17,18,19]. Both cell lines were cultured in cDMEM changing medium twice a week, were harvested via trypsin treatment. MSCs were used at passage 2.

Cells seeded at 2.0 × 10^5^ cells/well in six-well plates were treated with or without Ti particles (100 to 150 particles/mL in growth medium. Before assays cell cultures were washed with PBS three times to remove excess Ti particles and dead cells.

For in vitro cell commitment cells were seeded into 35-mm Petri dishes (50,000 cells/dish) and then cultured in differentiation media for 21 days: Adipogenic differentiation medium (StemMACS AdipoDiff Media, Miltenyi Biotec, Bergisch Gladbach, Germany); Osteogenic differentiation medium (StemMACS OsteoDiff. Media, Miltenyi Biotec).

In order to reproduce in vitro the inflammatory conditions present in case of peri-implantitis, cells were treated for 24 h with 0.1 mg /mL-1 of tumor necrosis factor alpha (Celbio), a well-defined inducer of inflammation, following the same conditions used in our previously work [20].

### 2.4. Methyl Hiazolyl-Tetrazolium (MTT) Assay

To determine the proliferation rate of cells in presence of Ti particles, the MTT-based cytotoxicity assay was performed according to the method of Denizot and Lang [21,22]. The test is based on mitochondrial viability, i.e., only functional mitochondria can oxidize MTT solution, yielding a blue-violet end product. After harvesting the culture medium, the cells were incubated for 3 h at 37 °C in 1 mL of 0.5 mg/mL MTT solution prepared in PBS. After removal of the MTT solution by pipette, 0.5 mL of 10% dimethyl sulfoxide in isopropanol was added for 30 min of incubation at 37 °C. For each sample, the absorbance values at 570 nm were recorded in duplicate of 200 μL aliquots deposited in 96-well plates using a multilabel plate reader (Victor 3, Perkin Elmer, Waltham, MA, USA). All samples were examined at 1, 3, and 7 days of treatment.

### 2.5. Reactive Oxygen Species (ROS) Measurements

The OxiSelect ROS Assay Kit (Cell Biolabs Inc., San Diego, CA, USA) is a cell-based assay for measuring the intracellular activity of hydroxyls, peroxyls, and other ROS. The assay employs the cell-permeable fluorogenic probe DCFHDA, which diffuses into cells and is deacetylated by cellular esterases into non-fluorescent DCFH. In the presence of ROS, DCFH is rapidly oxidized to highly fluorescent DCF. Fluorescence was read using a standard fluorometric plate reader [23].

### 2.6. Transmission Electron Microscopy (TEM)

The samples (60 peri-implantitis and 10 control samples) were preserved in a 2.5% glutaraldehyde/0.1 M sodium cacodylate buffer overnight at 4 °C. These samples were then treated with 1% OsO_4_/0.1 M sodium cacodylate buffer and dehydrated using ethanol solutions of increasing concentrations before being embedded in EPON™ epoxy resin. Ultrathin sections (Ultramicrotome, LKB, Stockholm, Sweden) were obtained and treated with 1% uranyl acetate and 1% lead citrate. The samples were analyzed by TEM (Electronic Microscopy Service, Department of Biology, University of Padova, Padova, Italy) using a Tecnai G12 electron microscope (FEI, Hillsboro, OR, USA, acceleration voltage 100 kV). The image acquisition system consisted of a Tietz video camera (Tietz Video and Image Processing Systems GmbH, Gauting, Germany) and TIA FEI imaging software 6 (FEI Company, Hillsboro, OR, USA).

### 2.7. Sample Preparation for Histological Analysis

The samples (20 peri-implantitis samples and 10 control samples) were fixed in 4% paraformaldehyde (Sigma-Aldrich) in PBS (EuroClone) for 24 h, and then dehydrated in a graded series of ethanol. After a brief rinse in xylene (Sigma-Aldrich), the samples were embedded in paraffin and cut into 5-μm-thick sections. The sections were placed onto poly-lysine-coated slides.

### 2.8. Hematoxylin and Eosin Staining

The sections were deparaffinized in xylene, rehydrated in graded concentrations of ethanol, and then stained with the nuclear dye hematoxylin (Sigma-Aldrich) and the counterstain eosin (Sigma-Aldrich). The sections were then rinsed, dehydrated in a graded series of ethanol and xylene, and coverslipped [24].

### 2.9. Immunofluorescence Staining

The sections were deparaffinized in xylene, rehydrated in a graded series of ethanol, and then incubated in 2% bovine serum albumin (BSA, Sigma-Aldrich) in PBS for 30 min at room temperature. The sections were then incubated with the primary antibodies in 2% BSA in a humidified chamber overnight at 4 °C. The following primary antibodies were used: rabbit polyclonal anti-human Von Willebrand factor antibody 1:100 (A0082, Dako, Milan, Italy); mouse monoclonal anti-CD31 antibody 10 μg/mL (ab24590, Abcam, Cambridge, UK); mouse monoclonal anti-FLK-1 antibody 1:100 (sc-6251, Santa Cruz Biotechnology Inc., Paso Robles, CA, USA); and mouse monoclonal anti-OB antibody 1:100 (sc-28344, Santa Cruz Biotechnology, Inc.). Immunofluorescence staining was performed for 1 h at room temperature with the secondary antibody DyLight 488-labeled anti-mouse IgG (KPL, Gaithersburg, MD, USA) or DyLight 549-labeled anti-rabbit IgG (H + L) (KPL) diluted to 1:1000 in 2% BSA. Nuclear staining was performed with 2 µg/mL Hoechst H33342 (Sigma-Aldrich) for 2 min. The sections were coverslipped with a drop of mounting medium.

### 2.10. RNA Extraction

Total RNA from the 60 peri-implantitis samples and 10 control samples was extracted using a RNeasy Mini Kit (Qiagen GmbH, Hilden, Germany), including DNase digestion with the RNase-Free DNase Set (Qiagen GmbH). The quality and concentration of the sample RNA were measured using a NanoDropTM ND-1000 (Thermo Scientific, Waltham, MA, USA).

### 2.11. RT2 Profiler PCR Array

For first-strand cDNA synthesis, 800 ng of total RNA from each sample (peri-implantitis tissue and healthy tissue) was reverse-transcribed with an RT2 First-Strand kit (Qiagen Sciences, Germantown, MD, USA). Real-time PCR (Polymerase chain reaction) was performed according to the user manual of the Human Wound Healing RT2 Profiler PCR array (SABiosciences, Frederick, MD, USA) using RT2 SYBR Green ROX FAST Mastermix (SABiosciences). The Human Wound Healing RT2 Profiler PCR array profiles the expression of genes related to the inflammation, granulation, and remodeling phases of wound healing. Thermal cycling and fluorescence detection were performed using a Rotor-Gene Q 100 system (Qiagen). The data were analyzed using Microsoft Excel-based PCR Array Data Analysis templates (SABiosciences). The results are reported as the relative expression (R) of each target gene in the peri-implantitis samples and control samples.

### 2.12. Real-Time PCR

For first-strand cDNA synthesis, 800 ng of total RNA from each sample (peri-implantitis tissue, healthy tissue, fibroblasts and MSCs) were reverse-transcribed with M-MLV Reverse Transcriptase (Invitrogen, Carlsbad, CA, USA), following the manufacturer’s protocol. Human primers were designed for each target gene with Primer 3 software (Table 1).

Real-time PCR was performed using the designed primers at a concentration of 300 nM and FastStart SYBR Green Master (Roche Diagnostics, Mannheim, Germany) on a Rotor-Gene 3000 device (Corbett Research, Sydney, Australia). The thermal cycling conditions were as follows: 15 min denaturation at 95 °C, followed by 40 cycles of 15 s of denaturation at 95 °C, 30 s of annealing at 60 °C, and 20 s of elongation at 72 °C. The expression level of cDNA samples was normalized to the expression of reference GAPDH.

### 2.13. GO Analyses

Genes found to be differentially expressed in in the peri-implantitis samples and control samples were analyzed with the GeneSpring Gene Ontology browser tool to identify the most represented gene ontology categories.

### 2.14. Array CGH Analysis

Array CGH was performed using Human Genome CGH 44K and 180K microarray kits (Agilent Technologies, Santa Clara, CA, USA). Labeling and hybridization were performed according to the supplier’s protocols. Briefly, 1 µg of purified DNA from 50 (20 healthy and 30 peri-implantitis) patient blood samples and 1 µg of pooled sex-matched reference DNA (Promega, Madison, WI, USA) were double-digested with RsaI and AluI for 2 h at 37 °C. After inactivation of the enzymes at 65 °C for 20 min, each digested sample was labeled by random priming (Genomic DNA Enzymatic Labeling Kit, Agilent Technologies) for 2 h using Cy5-dUTP for sample DNA and Cy3-dUTP for reference DNA. The labeled products were then column-purified (Microcon YM-30 filters, Millipore Corporation, Billerica, MA, USA). After probe denaturation and pre-annealing with Cot-1 DNA, hybridization was performed at 65 °C with rotation for 24 h. At the end of the incubation, the slides were washed and analyzed using an Agilent scanner. Data and graphical analyses were performed using CGH Analytics software (v3.1 Agilent Technologies). All map positions were based on the March 2006 NCBI 36/hg18 genome assembly [25].

### 2.15. Determination of Ti Levels by ICP-MS

The concentration of Ti in all samples were determined by inductively coupled plasma-quadrupole-mass spectrometry (ICP-QMS) using a model 7500cx instrument from Agilent Technologies (Tokyo, Japan). All reagents were purchased from Sigma-Aldrich, Milan, Italy. Samples were mineralized by acid digestion in an Ethos1 (Milestone) microwave oven. The digestion was carried out in Teflon vessels following a two-step program by adding to ~2 mg of the samples 10 mL of concentrated HNO_3_ (1st step at 200 °C, 50 min) and then 2 mL H_2_O_2_ 30% *w*/*w* (2nd step at 200 °C, 10 min). The digests of the residual dressings were centrifuged at 3000 rpm for 10 min to separate the AgCl precipitate formed, due to the presence of Cl^−^ in the cDMEM. The supernatant was collected and directly diluted in NH_4_OH 2.8% *w*/*w*, while the precipitate was dissolved in 1.5 mL of concentrated NH_4_OH (28% *w*/*w*) and then diluted in NH_4_OH 2.8% *w*/*w* for ICP-QMS analysis.

### 2.16. Statistical Analysis

One-way ANOVA was used for data analysis. Levene’s test was used to demonstrate equal variance in the variables. Repeated-measures ANOVA with Bonferroni’s multiple comparison post hoc analysis was performed. T-tests were used to determine significant differences (*p* < 0.05). Reproducibility was calculated as the standard deviation of the difference between measurements. All testing was performed using SPSS software, version 16.0 (SPSS, Inc., Chicago, IL, USA; licensed by the University of Padova, Italy).

## 3. Results

### 3.1. Effects of Ti Particles Exposure on Mitochondrial Function through ROS Production Shown by Decreased MTT Activity

In order to test if Ti nanoparticles could affect the main cells presents around the implants, i.e., fibroblasts and MSCs, cells were in vitro treated with a defined concentration of these nanoparticles. The physiology of the cells in both normal and inflammatory conditions was assessed, in order to mimic a peri-implantitis inflamed-like environment in vitro. At 1, 3, and 7 days, MTT assays were carried out to determine the mitochondrial function of cells treated with Ti particles (100–150 particles/cell). The principle of this test consists in the reduction of tetrazolium salts to formazan via mitochondrial reductase.

**Figure 2:**
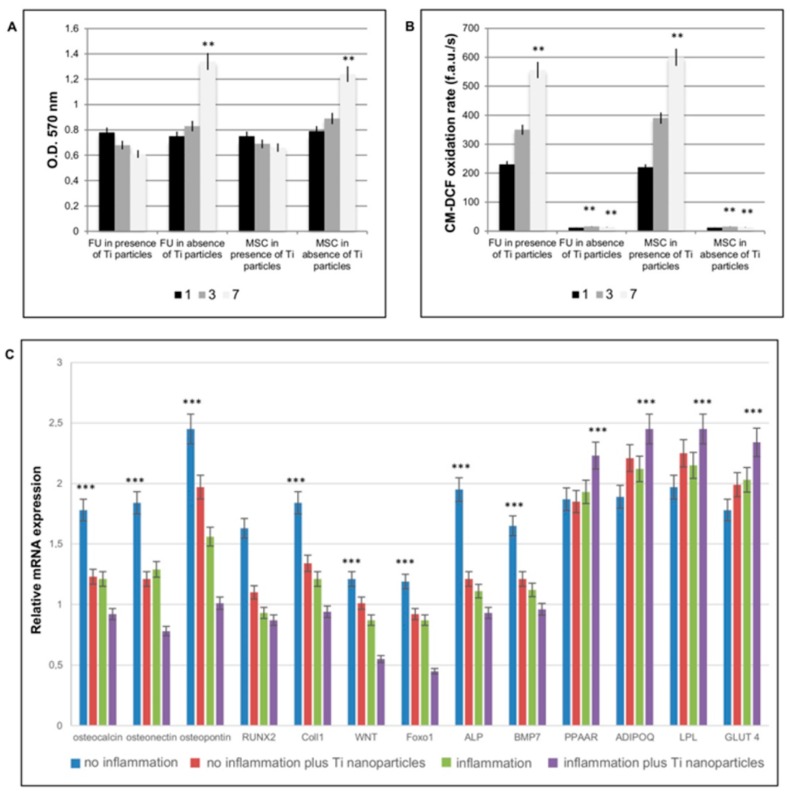
In vitro effect of titanium nanoparticles on fibroblastic and MSC cells. Effects of Ti particle exposure on mitochondrial function through ROS production assessed by MTT activity (A) and by evaluation of ROS production. The term “inflammation” is referred to in vitro conditions in which cells were treated with TNFa in order to reproduce the inflammatory conditions. (**A**) MTT assays at 1, 3, and 7 days, on fibroblasts (FU) and mesenchymal stem cells (MSCs) treated with or without Ti particles. (**B**) Effects of Ti particles on mitochondrial physiology evaluated by means of oxidation process activation in presence or absence of Ti particles. (**C**) Analyses of MSC commitment in presence of an inflammatory situation and of Ti nanoparticles. Genes related to an osteogenic commitment, such as osteocalcin, osteonectin, osteopontin, RUNX2, and Coll1; WNT; Foxo 1, ALP, and BMP7 show a decrease in expression in presence of inflammation and of Ti nanoparticles. Genes related to adipogenic commitment, such as PPARG, ADIPOQ, LPL, and GLUT4 show an increase in gene expression. The results for each experiment are from quadruplicate independent experiments. T-tests were used to determine significant differences (*p* < 0.05). * *p* < 0.05; ** *p* < 0.01; *** *p* < 0.001.

As shown in Figure 2A, a time-dependent decrease in MTT related to mitochondrial function activity was observed in both FU and MSCs treated with the Ti particles. Effects of Ti particles on mitochondrial physiology were also evaluated by means of oxidation process activation. Under environmental stress, such as the presence of disruptive bodies (i.e., Ti particles), cells react by increasing ROS generation, which leads to an imbalance between ROS generation and neutralization by antioxidative enzymes. This disturbance in the redox equilibrium is defined as oxidative stress. As shown in Figure 2B, the presence of Ti particles in both FU and MSCs induced a time-dependent increase in ROS production. In order to test if Ti nanoparticles could affect MSC commitment we cultured cells up to 21 days in presence of Ti nanoparticles and in inflammatory conditions. Gene expression related to the principal markers for osteogenic commitment such as osteocalcin, osteonectin, osteopontin, RUNX2, Coll1, WNT, Foxo1, ALP, BMP7 (Figure 2C) confirmed that, in the presence of inflammatory conditions, a decrease in osteogenic commitment occurred, as well as in presence of Ti nanoparticles. To note that the co-presence of inflammatory conditions and Ti nanoparticles enhanced this event. By contrary the adipogenic commitment (Figure 2C) detected by the presence of the adipogenic markers, such as PPAAR, ADIPOQ, LPL, and GLUT 4, confirmed that the inflammatory conditions enhanced the adipogenic commitment and that, also, in this case, the presence of Ti nanoparticles favored this process.

### 3.2. Evaluation of Ti in Peri-Implant Tissue

**Figure 3:**
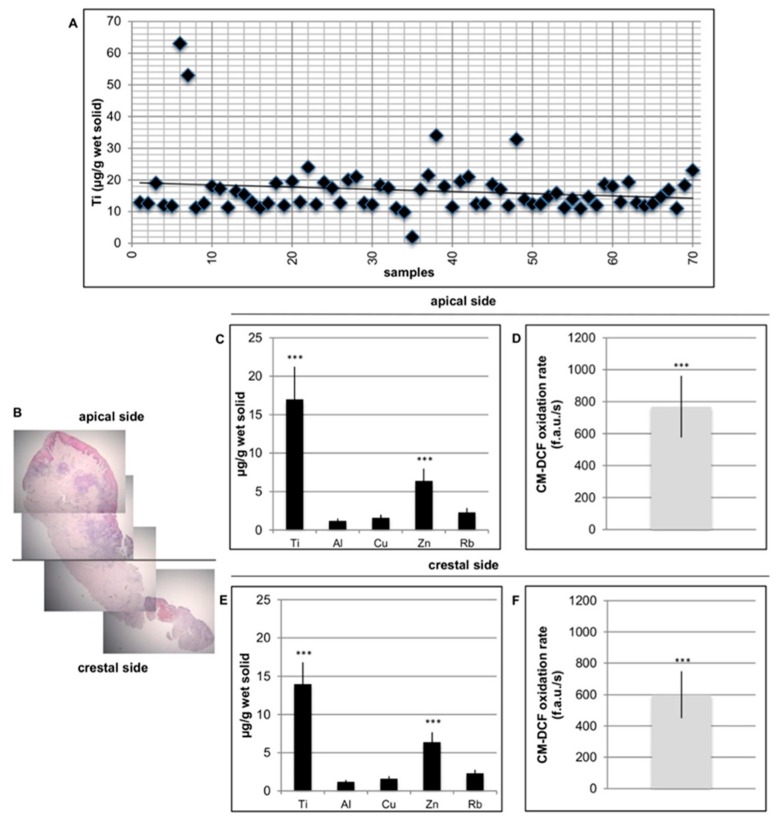
Detection of Ti in peri-implant tissue. (**A**) Ti level presence in the peri-implant tissue was detected in all the specimens. (**B**) Histological section of peri-implantitis affected sample related to the apical zone and to the crestal side. (**C**) Ti, Zn, Al, Cu, and Ru level in the apical side. (**D**) ROS levels in peri-implant tissue in the apical side. (**E**) Ti, Zn, Al, Cu, and Ru level in the crestal side. (**F**) Ti, Zn, Al, Cu, and Ru level in the crestal side. T-tests were used to determine significant differences (*p* < 0.05). * *p* < 0.05; ** *p* < 0.01; *** *p* < 0.001.

The level of Ti in the peri-implant tissue (healthy and peri-implantitis) was investigated in all the specimens. In the control samples, no Ti particles were detected (Figure 3A).

Together with Ti, other metals, including Zn, Al, Cu, and Ru, were evaluated both apically and crestally (Figure 3B). Additionally, in this case, on healthy tissue no significant presence of metal particles were identified (data not shown because the values are too slow). To evaluate whether metal particles could induce oxidative stress in cells, ROS levels in peri-implant affected tissue was analyzed. As reported in Figure 3B, well-defined values of ROS production were also present in this case on both sides of the specimens, apical and crestal. No significant levels of ROS in healthy tissues were found (data not shown because the values are too low).

### 3.3. Distribution of Ti Particles in Peri-Implant Tissue

**Figure 4:**
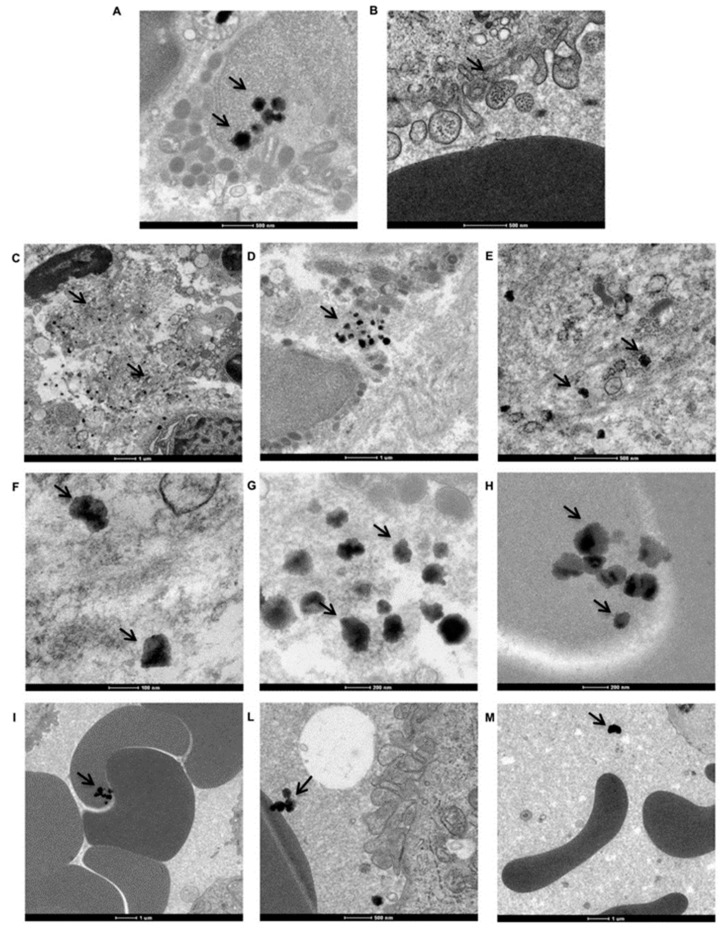
Distribution of Ti particles in peri-implant tissue. TEM imagines of Ti nanoparticles (black arrows) distribution on peri-implantitis affected tissue. (**A**) Ti particle agglomerates present inside the cells. (**B**) Ti particle agglomerates inside the cells via endocytic vesicles. (**C**–**E**) Ti particle aggregates present in the extracellular matrix (ECM). (**F**–**H**) Aggregates in different forms and sizes in ECM. (**I**–**M**) Ti particles associated with erythrocytes and plasma.

Ti nanoparticles were observed in all peri-implantitis affected specimens. In Figure 4 the most representative images obtained by TEM analyses are reported. The samples were observed from the apical side of the biopsy to the crestal side.

As shown in Figure 4A (black arrows), many Ti particle agglomerates were present inside the cells. In Figure 4B, it can be observed that Ti particle agglomerates entered the cells via endocytic vesicles. Upon examination of the same area at a higher magnification, aggregates are observed in the extracellular matrix (ECM) (Figure 4C–E, black arrows) and vary in both form and size (Figure 4F–H, black arrows). Moreover, Ti particles were observed in association with erythrocytes (Figure 4I–M, black arrows). Ti particles were observed within erythrocytes (Figure 4I, black arrows), attached to the outside of the erythrocyte membrane (Figure 4L), and simply dispersed in the extracellular environment (plasma, Figure 4M, black arrows).

### 3.4. Wound Healing Process

**Figure 5:**
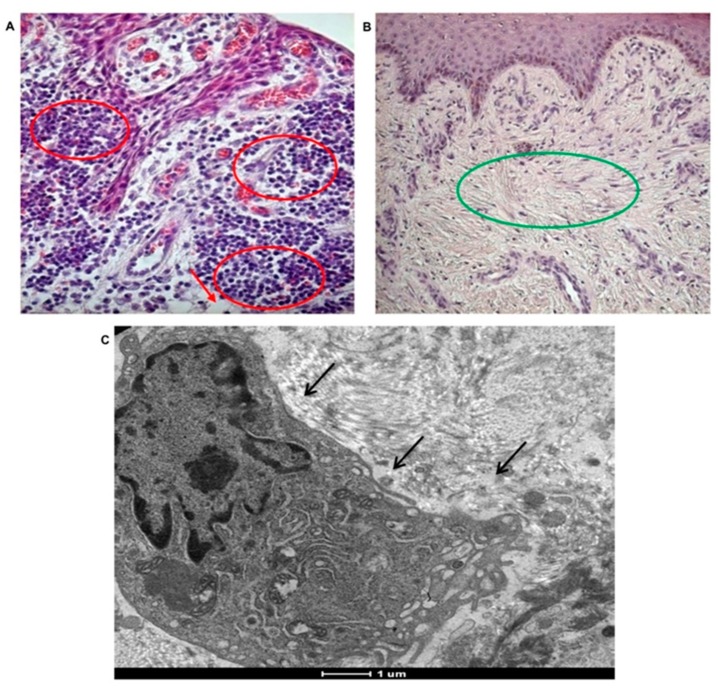
Morphological analyses of tissue affected by peri-implantitis. Presence of inflammation in tissues affected by peri-implantitis. (**A**) Histological staining of polymorphonuclear cells, mostly neutrophils, phagocytic cells (red circle) in tissues affected by peri-implantitis. Collagen fibers are not well represented. Adipose-like tissue (red arrows) is present (20× magnification). (**B**) Histological staining of healthy tissue showing no inflammatory event and well-organized collagen fibers (green circle). No adipose-like tissue is observable (10× magnification). (**C**) TEM imagine of extravasated macrophage, (black arrow) in tissues affected by peri-implantitis.

Histological staining of the peri-implantitis affected tissues confirmed the presence of inflamed tissue enriched with polymorphonuclear cells, mostly neutrophils, phagocytic cells (Figure 5A, black circles), inflamed cell population that was not detectable in the healthy tissue used as the control (Figure 5B).

In tissues affected by peri-implantitis the collagen fibers, forming the typical extracellular matrix on the sub-epithelium tissue are almost absent. The well-defined network of collagen fibers containing fibroblasts is evident in the control (Figure 5B green circle), while it is completely lost in in tissues affected by peri-implantitis. TEM analyses revealed the presence of extravasated macrophages, supporting the high phagocytic activity in case of peri-implantitis affected tissues (Figure 5C, black arrow).

**Figure 6:**
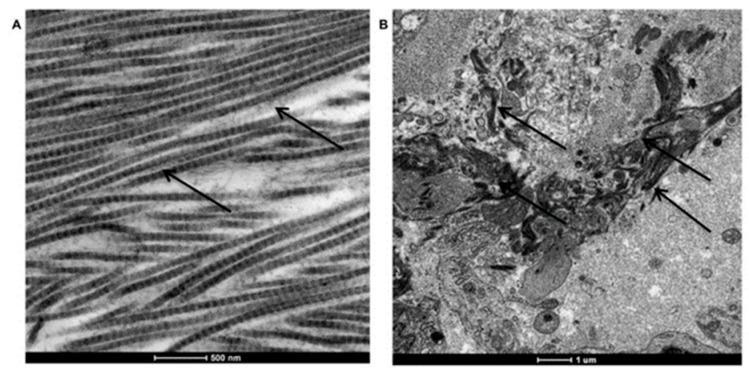
TEM analysis of collagen fiber disposition in peri-implant tissues. (**A**) Long and well-organized collagen fibers in healthy tissue. (**B**) Degraded and not organized collagen fibers in peri-implantitis affected tissue.

The presence of collagen fibers in peri-implant tissue was revealed also by TEM (control in Figure 6A and peri-implantitis-affected tissue in Figure 6B).

As reported, in healthy tissue collagen fibers are long and well organized (Figure 6A, black arrows); instead in peri-implantitis affected tissue collagen fibers are degraded, small in dimension and not organized (Figure 6B).

The wound healing process in peri-implantitis affected tissues was also analyzed in terms of the gene expression profile of remodeling enzymes, and inflammatory cytokines and chemokines.

**Figure 7:**
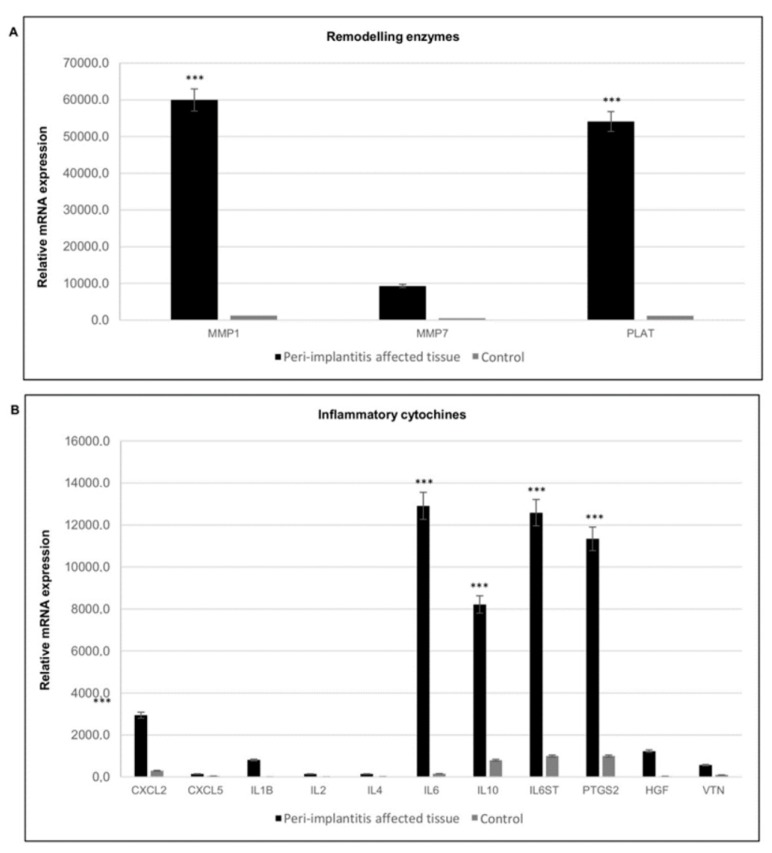
Gene expression profile of genes involved in extracellular matrix remodeling and of inflammatory cytokines. (**A**) Gene expression of matrix metallopeptidases in peri-implantitis affected tissues (black) and healthy tissue (grey). (**B**) Gene expression of inflammatory cytokines and chemokines metallopeptidases in peri-implantitis affected tissues (black), and healthy tissue (grey). The bars are related to the increase/decrease related to the value obtained from the control (peri-implant healthy tissue). Black bars peri-implantitis affected tissues, grey bars control. T-tests were used to determine significant differences (*p* < 0.05). * *p* < 0.05; ** *p* < 0.01; *** *p* < 0.001.

Considering the remodeling enzymes, significant up-regulation of matrix metallopeptidase 1 (MMP1) and MMP7 was detected (Figure 7A), in addition to high expression of tissue plasminogen activator (PLAT), which is a serine protease present in endothelial cells involved in the breakdown of blood clots. Plasminogen is transported to the wound by inflammatory cells early during the healing process, where it potentiates inflammation; high levels of plasminogen induce a delay in the resolution of inflammation and in the activation of the proliferation phase.

Gene expression analysis of inflammatory cytokines and chemokines in peri-implant tissues showed high expression of inflammatory mediators, such as interleukin 6 (IL6) and IL1B, but also an increase in an anti-inflammatory one such as IL10, in addition to chemokine (C-X-C motif) ligand 2 (CXCL2). Conversely, CXCL5 showed lower transcript levels. This chemokine activates neutrophils, mainly during an acute inflammatory response. Elevated expression of hepatocyte growth factor (HGF) and the osteoclast-specific protein vitronectin (VTN) was also detected (Figure 7B).

### 3.5. Osteogenesis and Adipogenesis

**Figure 8:**
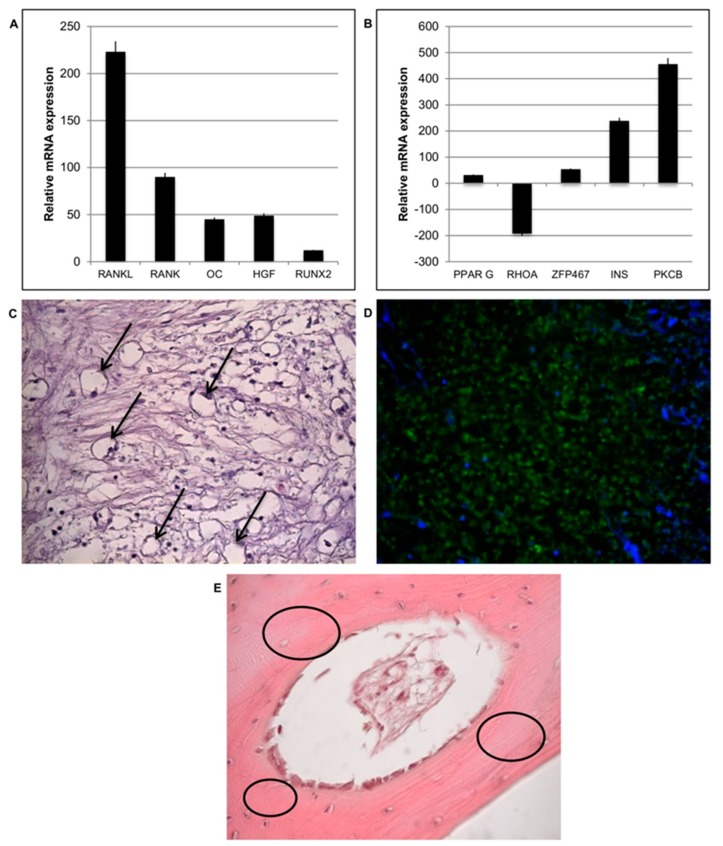
Osteogenesis and adipogenesis events during peri-implantitis. (**A**) Gene expression of most significant genes related to osteogenesis is down regulated in peri-implantitis affected tissue compared to that of the control. (**B**) Gene expression of most significant genes related to adipogenesis are up regulated in peri-implantitis affected tissue compared to that of the control. (**C**) Histological analysis of peri-implantitis affected tissue: presence of adipocytes (black arrows) (20× magnification). (**D**) Positive staining of a hormone related to adipose tissue such as Leptin (green) and nuclei (blue) confirmed the presence of adipose-like cells; (20× magnification) (**E**). Histology of control healthy tissue: no adipose tissue is present. A well-organized osteon structure (black circle) is present.

The balance between osteogenesis and adipogenesis in the peri-implantitis affected samples was analyzed. Osteogenesis is the biological event driving the differentiation of MSCs into osteoblasts and then into osteocytes; this process was evaluated in the peri-implantitis affected tissues by means of gene expression profiling. Regarding the expression of osteogenic markers, a very slow gene expression of runt-related transcription factor 2 (RUNX2) and osteocalcin (OC) was observed (Figure 8A). By contrast, tumor necrosis factor ligand superfamily member 11 (RANKL) was up-regulated at high levels. The expression of tumor necrosis factor receptor superfamily member 11a NFKB activator (RANK) was low. This indicates a reduction in osteogenesis and an increase in osteoblastogenesis in peri-implantitis affected tissue.

Adipogenic event is activated on peri-implantitis affected tissue: expression of peroxisome proliferator activated receptor gamma (PPARG) and insulin (INS), which regulates fatty acid storage, glucose metabolism and adipogenesis by fat cells, was detected (Figure 8B) and it is up regulated. The up-regulation of zinc finger protein 467 (ZFP467), which is a regulator of osteoblast and adipocyte commitment, was also evident indicating that a switch between osteogenic and adipogenic phenotypes had begun. Further confirming the presence of atypical soft matrices at the contact site, it was also observed a strong down-regulation of Ras homolog gene family, member A (RHOA), a specific protein strongly related to mechano-transduction activity (Figure 8B). It seems that the loss of a strong extracellular environment due to an adipogenic phenotype induces a down regulation of this gene. In addition, high values of protein kinase C beta (PKCB), which is involved in adipogenesis, was detected in the presence of oxidative stress. The presence of cells with an adipocyte-like morphology in the peri-implantitis affected tissues was also revealed by classical histological staining (Figure 8C). Cells with the typical adipocyte phenotype, characterized by a round shape, basal nuclei, and extensive white cytoplasm, were observed (Figure 8C, black arrows). Adipose tissue is completely absent in the healthy bone tissue (control) (E), where indeed a well-defined bone matrix is present (black circle). Immunofluorescence staining (Figure 8D, green) was performed to detect the adipose hormone leptin. Leptin was observed throughout the peri-implantitis affected tissues, confirming the ability of cells to secrete this hormone. The negative staining (data not shown) for this hormone in control confirmed that in normal condition there is the presence of nature bone tissue.

### 3.6. Vascularization

**Figure 9:**
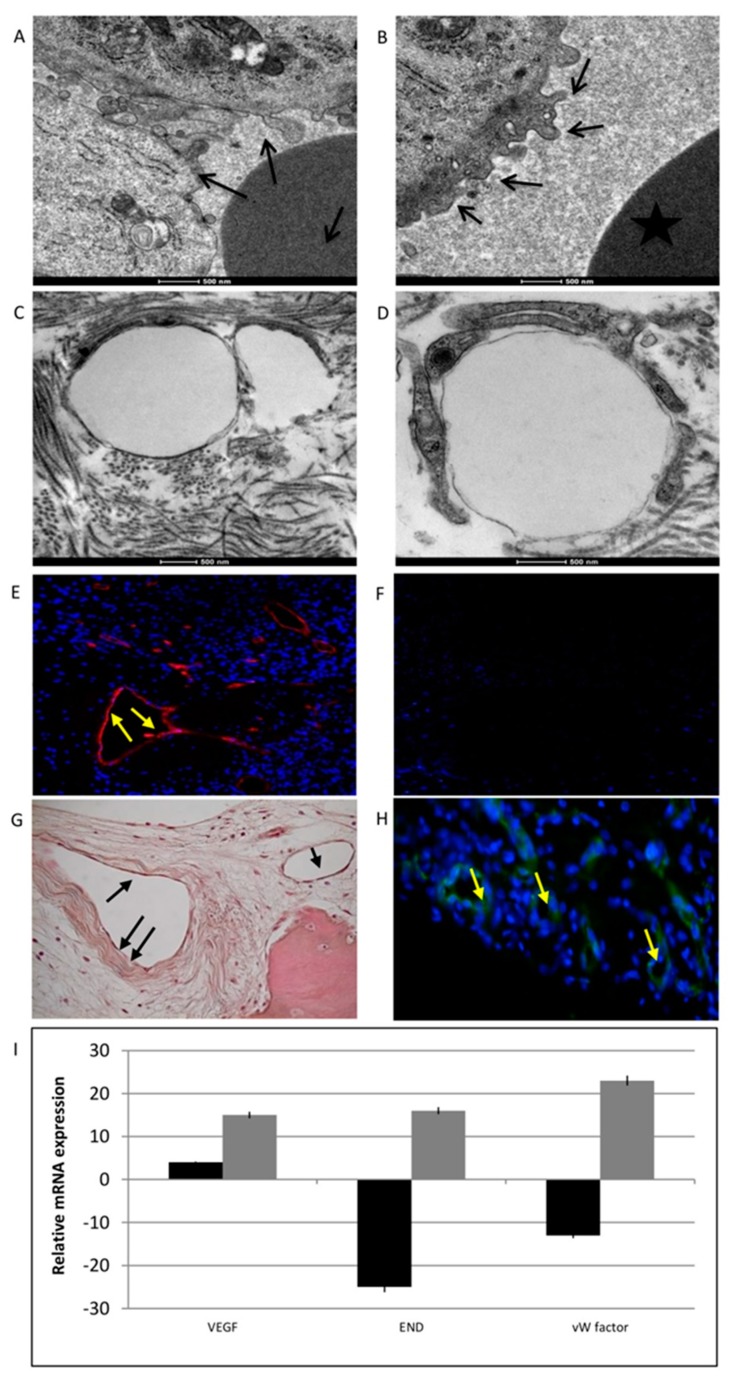
Analyses of vascularization process. (**A**,**B**) TEM analyses of peri-implantitis affected tissues showing endothelial cells with irregular membranes and abnormal bubbles (black arrows). (**C**,**D**) TEM analyses of endothelial cells in control samples. (**E**) Immunofluorescence staining of VWF (red) and nuclei (blue) in peri-implantitis affected tissue and in control (**G**). (**F**) Immunofluorescence against CD31. No signal indicates the absence of mature endothelial cells in peri-implantitis affected tissue and in control (**H**) No signal confirms the absence of mature endothelial cells. (**I**) Gene expression analyses of the markers related to vascularization: VEGFA, ENG, and VWF. Black bars peri-implantitis affected tissue, grey bars control, results are related to the up-regulation or down-regulation comapre to the house keeping. T-tests were used to determine significant differences (*p* < 0.05). * *p* < 0.05; ** *p* < 0.01; *** *p* < 0.001.

Blood vessels in the peri-implantitis affected tissues were investigated by microscopy and gene expression profiling. Morphological analysis of the peri-implantitis affected tissues by TEM revealed irregular endothelial membranes with abnormal bubbles (Figure 9A,B, black arrows) compared to the normal, smooth, and well-defined endothelial membranes present on healthy tissue—control (Figure 9C,D, black arrows).

Endothelial cell markers were detected by immunofluorescence against von Willebrand Factor (VWF), CD31, and FKL-1 (alias VEGF receptor 2). Positive staining for VWF was observed in peri-implantitis affected tissue (Figure 9E, yellow arrows) and in control (Figure 9G, black arrows). This glycoprotein plays a role in the hemostasis; it is constitutively produced by endothelial cells and stored in Weibel-Palade bodies. However, the marker of mature endothelial cells, CD31 (Figure 9F for peri-implantitis affected tissue and H for control, yellow arrows), was not detected in the case of peri-implantitis. This atypical behavior of endothelial phenotype was also confirmed at the mRNA level. Gene expression analyses of markers related to vascularization, such as VEGFA, endoglin (ENG), and VWF, revealed low VEGFA transcript levels and the down-regulation of END and VWF (Figure 9I: black bars for peri-implantitis affected tissue, grey bars for control).

### 3.7. Gene Ontology (GO) of Genes

**Figure 10:**
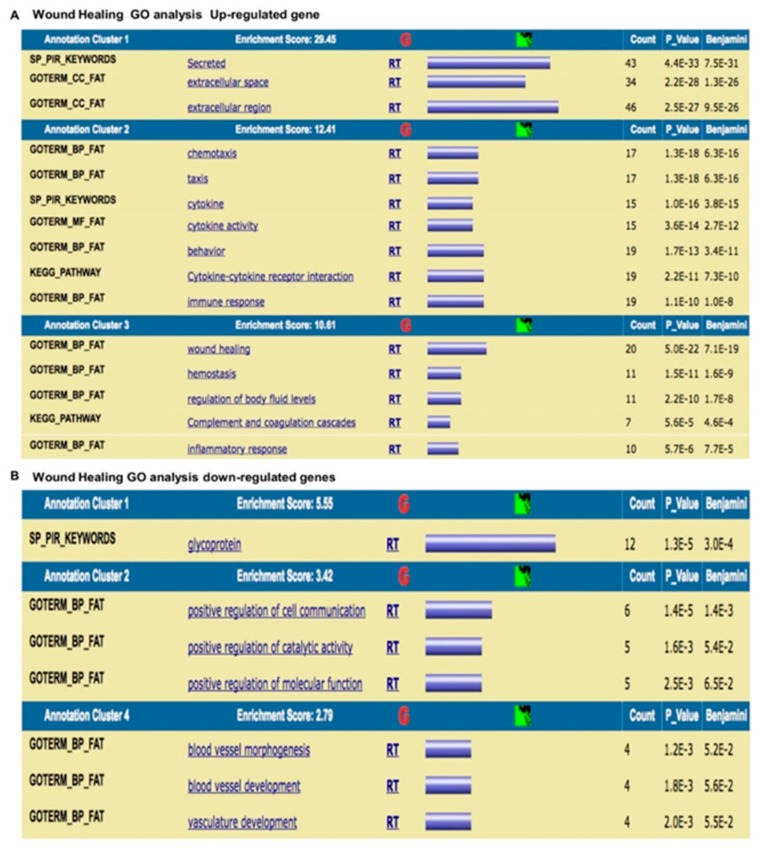
Gene Ontology analysis of genes related to the wound healing process. (**A**) Up-regulated genes are related to the wound healing process. (**B**) Down-regulated genes are involved in cell signaling/migration and blood vessel morphogenesis.

**Figure 11:**
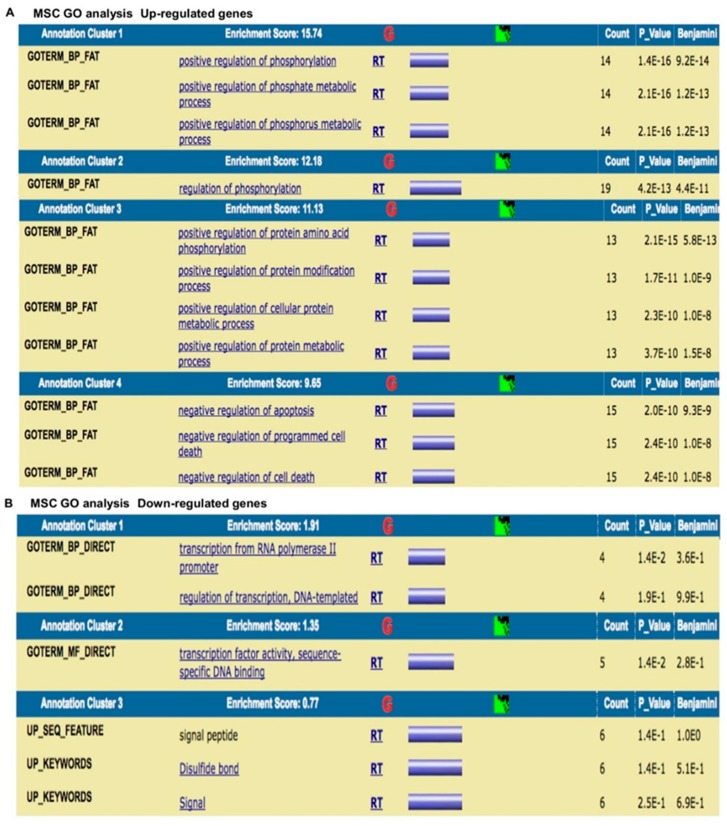
Gene Ontology analysis of genes related to MSCs involved in tissue homeostasis. (**A**) Up-regulated genes are involved in intense kinase activity. (**B**) Down-regulated genes are involved in transcription process.

The expression of genes related to the wound healing process and to MSCs involved in tissue homeostasis was evaluated, and differentially expressed genes were found in the peri-implantis affected tissue compared with the controls (peri-implant healthy tissue). The results were analyzed using the GeneSpring GO browser tool to identify the most represented GO categories in the down-regulated genes (Figure 10 and Figure 11).

The results were as follows: (a) up-regulated genes related to the wound healing process are involved in intense cellular activity related to molecule secretion, immune responses, blood coagulation and wound healing (Figure 10A); (b) down-regulated genes are involved in cell signaling/migration and blood vessel morphogenesis (Figure 10B); (c) up-regulated genes related to MSCs are involved in intense kinase activity (Figure 11A); and (d) down-regulated genes are related to MSCs are involved in transcription process (Figure 11B).

### 3.8. Chromosomal Aberration

**Figure 12:**
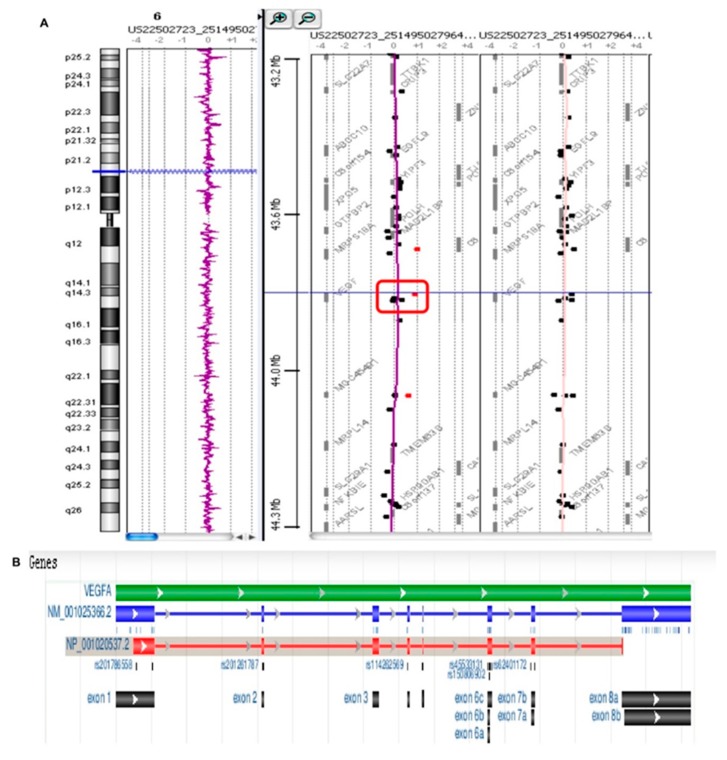
Array CGH analysis. (**A**) Patients affected by peri-implantitis show a heterozygote duplication in a genome portion corresponding to a region of chromosome 6, starting at the 43846044 bp position and stopping at 43862079 bp for a total of approximately 16 kb (red square). (**B**) This duplication was not detected in the control group (patients not affected by peri-implantitis)

Array CGH was used to analyze DNA derived from the blood samples collected from patients affected by peri-implantitis and in healthy patients. The results showed that all patients affected by peri-implantitis had a heterozygote duplication of a genome portion corresponding to a region of chromosome 6, starting at the 43,846,044 bp position and stopping at 43,862,079 bp, for a total of approximately 16 kb (Figure 12A, red square). This duplication was not detected in the control group (patients not affected by peri-implantitis) (Figure 12B). Further analyses of this genomic portion revealed that this position corresponded to exon 6 of the VEGFA gene (Figure 12B).

## 4. Discussion

The aim of the present study was to offer a new approach based on the analysis of the effects of Ti nanoparticles in inducing a chronic inflammatory state in peri-implant disease.

Previous analyses have been focused on metal debris-induced osteolysis after orthopedic surgery, which is a major cause of aseptic implant loosening [26,27,28,29,30,31,32,33,34,35,36]. Different cell types, including macrophages, monocytes, osteoblasts, and osteoclasts, are involved in this process. Recently, Heleem Smith et al. [37] showed that MSCs/osteoprogenitor cells are another related target and that the endocytosis of Ti particles reduces MSCs proliferation and osteogenic differentiation.

Based on these reports, we analyzed the in vitro effect of Ti particles on fibroblasts and MSCs and their subsequent distribution within peri-implant tissues. Our results confirm that in vitro, Ti particles can affect mitochondria and induce the production of ROS. We detected the presence of Ti in all peri-implant (or peri-implantitis affected) tissue specimens. Ti particles were present in different sizes and forms and were observed within cells (internalized by endocytosis), in the ECM, inside vessels, dispersed in plasma, on erythrocyte membranes and within erythrocytes. Additionally, ROS production was detected in all specimens. ROS are strongly related to neutrophil recruitment, an event that is a central aspect of the inflammatory process and critical for clearing a variety of pathogens. The number of neutrophils at sites of infection must be tightly regulated to ensure that sufficient neutrophils are recruited for efficient clearance while minimizing excess recruitment, which drives immune pathology [38]. The mechanisms that define the optimum number of neutrophils at inflammation sites are unknown. Neutrophil recruitment is regulated by pro-inflammatory cytokines, such as CXCL2; [39,40,41] we found that this molecule was expressed in the peri-implant (or peri-implantitis affected) tissues. Recent work has implicated neutrophils in IL1B production upon bacterial infection, [42,43,44] suggesting that neutrophils could play more central roles in modulating inflammation. Compared with other phagocytes, neutrophils generate higher concentrations of ROS [45,46,47]. In addition to their cytotoxic role, ROS regulate cell signaling [48] and inhibit inflammasome activation and cytokine expression [49,50,51,52,53,54,55,56]. However, the physiological purpose of this oxidative regulatory mechanism remains unknown. Warnatsch et al. [57] used microbes of various sizes to demonstrate that the ability to sense ROS localization allows neutrophils to adjust inflammation by modulating their own recruitment. In our study, Ti particles affected the resident cells by inducing ROS generation that could be responsible for the recruitment of neutrophils. These particles, due their non-degradability, exert a continuous effect on neutrophil recruitment.

In the peri-implantitis affected samples, it was possible to observe an excess of macrophages and neutrophils, contributing to an impaired inflammatory phase. The existence of an inflammatory response in these tissues was also confirmed by observing an increased mRNA level of prostaglandin-endoperoxide synthase (PTGS2), an enzyme expressed only in inflamed tissues [58]. The inflammatory response is shaped by multiple cytokines and chemokines, which arise mainly from resident fibroblasts, keratinocytes, and endothelial cells. As expected, the presence of inflammatory cytokines and chemokines, particularly IL1B, IL6, its signal transducer IL6ST, and CXCL2, was clearly observed at the mRNA level in the peri-implantitis affected samples. IL1B is released by macrophages together with TNF and IL6 to promote the recruitment of other inflammatory cells [59]. Interestingly, the achievement of critical concentrations of pro-inflammatory mediators has been reported to stimulate inflammatory osteoclastogenesis, which is orchestrated by the interaction of RANK with its ligand RANKL [60]. Hengartner and colleagues showed that the prolonged local secretion of IL1B was associated with severe bone loss and delayed fracture healing [61,62,63,64,65,66,67].

In the present study, several MMP, such as MMP2, MMP7, and MMP9, were strongly up-regulated in the peri-implantitis affected samples. MMP are responsible for remodeling and degrading ECM molecules by cleaving components of cell-cell junctions and cell-matrix contacts [68]. The high production of MMP that we observed, together with the reduced expression of their inhibitor TIMP1, could be associated with tissue destruction responsible for the generation of a very soft peri-implant environment. In contrast, a hard ECM environment is required for the osteoblastic commitment of MSCs because they are responsive to mechanical stimulation. This sensing mechanism depends on actomyosin contractility, which is largely controlled by the RHOA/ROCK pathway. RHOA influences myosin-generated cytoskeletal tension and it is important in determining MSC commitment to the adipocyte or osteocyte lineages [69]. In particular, the down-regulation of RHOA causes adipogenesis, whereas its activation promotes osteogenesis [70,71]. To verify whether RHOA down-regulation could be associated with an adipogenic process in the peri-implantitis affected samples, a selected panel of adipogenic and osteogenic markers were profiled by real-time PCR. The down-regulation of RHOA seemed to favor the commitment of MSCs to the adipocyte lineage, as confirmed by the up-regulation of the master adipogenic transcription factor PPARG and the down-regulation of RUNX2, which drives the commitment of MSCs to the osteoblast lineage.

In the peri-implantitis affected tissues, we found that WNT5A expression was strongly down-regulated and that ZFP467 mRNA was up-regulated. ZFP467 is a co-factor that promotes adipocyte differentiation and suppresses osteoblast differentiation [70,71,72] as well as the increase of PKCB [73]. Histological evaluation of tissues with peri-implantitis supported the gene expression data discussed above. Indeed, cells with a typical round shape, cytoplasm pushed toward the cell edge, and a flattened nucleus along the cell membrane were observed in the peri-implantitis tissues. The adipocyte phenotype was further confirmed by positive staining for leptin, as well as high INS mRNA expression. These finding are in accord with those of numerous studies suggesting that insulin is an inducer of leptin expression [74]. Leptin is an adipokine produced by fat cells that influences a variety of physiological events, such as energy homeostasis and bone remodeling [75]. The connection between energy utilization and skeletal turnover begins in the bone marrow with the lineage allocation of MSCs to adipocytes or osteoblasts. Mature bone cells secrete OC, which influences insulin sensitivity, and fat cells synthesize leptin, which regulates osteoblast differentiation. Leptin decreases osteoblast activity and bone formation and increases bone resorption via RANKL production, leading to vertebral trabecular bone loss [76]. These data, together with the down-regulation of RUNX2, which drives the commitment of MSCs to the osteoblast lineage, provide evidence that an increase in the osteoclast population over the osteoblast population occurs in case of peri-implantitis. Our results are in agreement with the observations of several other authors, such as Schminke et al., [77] who recently found that RUNX2 is decreased in tissues affected by peri-implantitis.

The results obtained in this study suggest a possible correlation between the alterations of the vascularization process and the osteolytic events that occur in peri-implantitis disease.

Another biological event we investigated was angiogenesis, because the GO analyses provided evidence of vessel formation down-regulation. TEM analyses revealed abnormal features at the membrane level and cell junction disturbances. The impairment in the vascular organization of tissues affected by peri-implantitis was also analyzed at the molecular level. ENG mRNA was present at low levels, and CD31 protein was not detected in the peri-implantitis samples. The functions of ENG are linked to angiogenesis and endothelial cell differentiation, adhesion, and migration, as well as to maintaining the homeostasis of vascular walls [78]. The glycoprotein CD31 plays a key role in adhesion between endothelial cells and in the interactions of these cells with leukocytes [79,80,81,82] Vascular endothelial cells adhere to each other through specialized cell-cell junctions, such as adherent junctions.

Genetic analyses performed by array CGH on subjects affected by peri-implantitis revealed the amplification of the gene encoding VEGFA. Of the seven members composing the VEGF protein family, VEGFA is the most abundant form and is, therefore, commonly used in studies investigating the biological effects of VEGF [82,83,84,85,86,87]. In 2010, Mierzwinska-Nastalska et al. [88] investigated the concentrations of VEGF protein in gingival crevicular fluid in healthy and diseased soft tissues surrounding implants. Higher VEGF concentrations were found in crevicular fluid around the implants than in the clinically healthy sites. Di Alberti and coworkers evaluated the VEGF mRNA levels in bone around healthy and failing dental implants. Their results demonstrated that VEGF may be important for regulating tissue healing and bone remodeling in peri-implantitis lesions [89]. The work of Street and colleagues confirmed the key role played by VEGF in bone remodeling through the stimulation of alkaline phosphatase activity and osteoblast differentiation [90]. In particular, they demonstrated that VEGF inhibition dramatically inhibited the healing of a tibial cortical bone defect and that exogenous VEGF enhanced blood vessel formation, ossification, and new bone maturation in mouse femur fractures. Furthermore, Hu and Olsen confirmed the important role of osteoblast-derived VEGF in bone healing using transgenic mice with the osteoblast-specific deletion of VEGF [91,92,93] As reported in an article by Yang et al., [92] several other studies have confirmed the role of VEGF in ossification. They confirmed that osteogenesis and angiogenesis are closely correlated processes. During these processes, VEGF acts as an essential mediator not only in bone angiogenesis, but also in various aspects of bone development.

The study has some limitations. First, a convenience sample was used because the study was design as hypothesis generator, and the sample size of each test was driven by practical reasons (including costs, time, staff workload, and resources). Further research is needed to demonstrate the findings suggested in this analysis.

## 5. Conclusions

In conclusion, our analysis of tissues affected by peri-implantitis revealed correlations between the presence of Titanium nanoparticles and ROS production and we can speculate that the event occurring at the implant site can be summarized as follows:

1. Metal particles may induce ROS production.
2. ROS production may induce abnormal neutrophil recruitment as metal particles are not degradable.
3. Neutrophils induce ECM degradation through the secretion of MMP.
4. ECM resistance changes, due its degradation, inducing the mechano-transduction of MSCs differentiation more toward adipocytes than osteoblasts.
5. ROS induce the activation of PKC beta, which is involved in MSC commitment to the adipocyte lineage.
6. VEGF involvement in bone regeneration is down-regulated due to a genetic deletion.
7. Osteolytic processes are activated, due to an imbalance of osteogenic commitment.

Limitations of the present study include the variability of the implant surfaces and the characteristics of the control group, represented by healthy mucosal specimen retrieved 2–3 months after implant insertion and not after a time equivalent to that spent for the onset of peri-implantitis. Findings from this work provide pilot data for future studies, in which patients restored with the same type of implants will be recruited. 

## Figures and Tables

**Table 1:** Human primer sequences. ENG (endoglin), INS (insulin), OC (osteocalcin), OPG (osteoprotegerin), OPN (osteopontin), PPARG (peroxisome proliferator-activated receptor gamma), RANK (tumor necrosis factor receptor superfamily, member 11a, NFKB activator), RANKL (tumor necrosis factor ligand superfamily, member 11), RHOA (Ras homolog gene family, member A), VEGF (vascular endothelial growth factor), VWF (von Willebrand factor), ZFP467 (zinc finger protein 467), and GAPDH (glyceraldehyde 3-phosphate dehydrogenase).

Gene	FORWARD (5′ → 3′)	REVERSE (5′ → 3′)	Product Length
ENG	TGTCTCACTTCATGCCTCCAG	GCTCTTTCTTTAGTACCAGGGTCA	161 bp
INS	AGGCTTCTTCTACACACCCAAG	CGTCTAGTTGCAGTAGTTCTCCA	199 bp
OC	GCAGCGAGGTAGTGAAGAGAC	AGCAGAGCGACACCCTA	193 bp
OPG	AAACGCAGAGAGTGTAGAGAGG	TCGAAGGTGAGGTTAGCATGTC	183 bp
OPG	TGGAAAGCGAGGAGTTGAATGG	GCTCATTGCTCTCATCATTGGC	192 bp
PPARG	CAGGAGATCACAGAGTATGCCAA	TCCCTTGTCATGAAGCCTTGG	173 bp
RANK	GATCGGTACAGTCGAGGAAGA	TGCTGCGAGTTTGAGGAGTG	169 bp
RANKL	TCAGCATCGAGGTCTCCAAC	CCATGCCTCTTAGTAGTCTCACA	194 bp
RHOA	TGGACTCGGATTCGTTGCC	ACCTGCTTTCCATCCACCTC	183 bp
VEGF	GGACAGAAAGACAGATCACAGGTAC	GCAGGTGAGAGTAAGCGAAGG	182 bp
VWF	GCTTCACTTACGTTCTGCATGA	CCTTCACTCGGACACACTCATTG	174 bp
ZFP467	CGCTGAGCTGAAGTTCTTGGA	ACCACTCTTTCCTGCCCTG	102 bp
GAPDH	TCAACAGCGACACCCAC	GGGTCTCTCTCTTCCTCTTGTG	203 bp
Collagen type 1	TGAGCCAGCAGATCGAGA	ACCAGTCTCCATGTTGCAGA	128 bp
RUNX 2	CGTGGATCCATGGCT	CCTCGATCGAAGGACT	102 bp
wnt	CAGGAGATCACAGAGTATGCCAA	TCCCTTGTCATGAAGCCTTGG	132 bp
FOXO1	GACAAGTACAAGCTGAGCAAGAAG	CCACAAGCACCACATACTCCTG	163 bp
ALP	TCAGAGGGAAGGAGATAGAGAGTC	AGCCAGAAACCATATGTCAAGAGA	112 bp
BMP7	AGATGCGGTGGCTAAAGGTC	TCTTAGGCAGCTCTTTGGGA	145 bp
ADIPOQ	GATGAGAGTCCTGGGTGTGAG	CTGGGTAGATATGGGATTCAAGAGA	151 bp
LPL	CAGCAAGAGCAAGGAGAAGAAAC	GTGGTAGGTGATGTTCTGGGA	184 bp
GLUT 4	CCTGATCATTGCGGTCGTG	CCGAGACCAAGGTGAAGACTG	122 bp
